# *Mycobacterium tuberculosis *PPD-induced immune biomarkers measurable *in vitro *following BCG vaccination of UK adolescents by multiplex bead array and intracellular cytokine staining

**DOI:** 10.1186/1471-2172-11-35

**Published:** 2010-07-07

**Authors:** Steven G Smith, Maeve K Lalor, Patricia Gorak-Stolinska, Rose Blitz, Natalie ER Beveridge, Andrew Worth, Helen McShane, Hazel M Dockrell

**Affiliations:** 1 Department of Infectious and Tropical Diseases, London School of Hygiene & Tropical Medicine, Keppel Street, London WC1E 7HT, UK; 2 The Jenner Institute, Old Road Campus Research Building, Roosevelt Drive, Oxford, UK

## Abstract

**Background:**

The vaccine efficacy reported following *Mycobacterium bovis *Bacillus Calmette Guerin (BCG) administration to UK adolescents is 77% and defining the cellular immune response in this group can inform us as to the nature of effective immunity against tuberculosis. The aim of this study was to identify which cytokines and lymphocyte populations characterise the peripheral blood cellular immune response following BCG vaccination.

**Results:**

Diluted blood from before and after vaccination was stimulated with *Mycobacterium tuberculosis *purified protein derivative for 6 days, after which soluble biomarkers in supernatants were assayed by multiplex bead array. Ten out of twenty biomarkers measured were significantly increased (p < 0.0025) 1 month after BCG vaccination when compared to paired samples (n = 12) taken prior to vaccination (IFNγ, TNFα, IL-1α, IL-2, IL-6, IL-10, IL-17, GM-CSF, MIP1α, IP-10). All of these remained detectable by multiplex bead array in samples taken 12 months after BCG vaccination of a partially overlapping adolescent group (n = 12). Intracellular cytokine staining after 24 hour *Mycobacterium tuberculosis *purified protein derivative stimulation of PBMC samples from the 12 month group revealed that IFNγ expression was detectable in CD4 and CD8 T-cells and natural killer cells. Polyfunctional flow cytometry analysis demonstrated that cells expressing IFNγ alone formed the majority in each subpopulation of cells. Only in CD4 T-cells and NK cells were there a notable proportion of responding cells of a different phenotype and these were single positive, TNFα producers. No significant expression of the cytokines IL-2, IL-17 or IL-10 was seen in any population of cells.

**Conclusions:**

The broad array of biomarker responses detected by multiplex bead array suggests that BCG vaccination is capable, in this setting, of inducing a complex immune phenotype. Although polyfunctional T-cells have been proposed to play a role in protective immunity, they were not present in vaccinated adolescents who, based on earlier epidemiological studies, should have developed protection against pulmonary tuberculosis. This may be due to the later sampling time point available for testing or on the kinetics of the assays used.

## Background

That the cytokine interferon gamma (IFNγ) plays an important role in the protective immune response against tuberculosis (TB) is indicated by the susceptibility of mice and humans with IFNγ signalling pathway deficiencies to TB disease [1-3]. Its detection in isolation however is not a sufficient indicator of a protective immune phenotype as those with latent infection and a positive IFNγ release assay status can progress to active disease and IFNγ secretion can also be detected in samples from patients with active disease [4].

The Th-1-type immune response that is most effective against TB and of which IFNγ is a component is likely to include other cytokines such as tumour necrosis factor alpha (TNFα), interleukin (IL) -2 and IL-12. A role for the more recently identified Th-17 phenotype involving IL-17 has also been described [5,6]. Furthermore, the presence of cells that secrete such cytokines as well as other immune effector molecules may be included as a component of a protective biomarker profile. For example, CD4^+ ^T-cells play an important role in TB immunity, however CD8^+^, NKT and γδ T-cells may also be necessary [7-10]. A protective biomarker signature may also be defined by the absence of particular biomarkers as certain immune states may subvert the response to TB, allowing bacterial infection to persist and for disease to eventually progress. Cytokines such as IL-4 or other immunoregulatory cytokines such as IL-10 derived from Th-2 biased T-cells or regulatory T-cells respectively may indicate such a subversion if detected [11,12].

BCG vaccination has previously demonstrated a protective efficacy of 77% against pulmonary tuberculosis when administered to UK adolescents [13]. We have used this setting to investigate the nature of the immunity induced by BCG vaccination in representative cohorts of UK schoolchildren (age range 12-15). Diluted whole blood assays on samples from such a cohort, in which responses to antigen during 6 day cultures were measured by quantifying IFNγ in assay supernatants, revealed increased IFNγ after vaccination compared to that measured in pre-vaccination samples [14]. The status of IFNγ as a cytokine that is necessary but not sufficient for protection against TB is illustrated by parallel experiments carried out in Malawi where high concentrations of IFNγ were detected both prior to and following BCG vaccination in a setting where BCG is much less protective than in the UK [14]. Furthermore, studies have described other biomarkers that can differentiate latent infection from active disease [15] and have highlighted the importance of cytokines such as TNFα [5,16]. Such observations emphasise the need to measure a greater diversity of potential biomarkers in order to develop a more detailed representation of the BCG-induced immune response in different settings. In South Africa, studies on samples from BCG-vaccinated infants to look at responses comprising the Th-1 cytokines IFNγ, TNFα and IL-2 revealed multiple T-cell phenotypes with distinct cytokine secretion profiles [17]. Our group has also recently reported extensive and complex cytokine responses measurable in *Mycobacterium tuberculosis *purified protein derivative (*Mtb *PPD)-stimulated blood from BCG-vaccinated, UK infants [18]. New candidate TB vaccines that are thought to represent more efficacious alternatives to BCG have demonstrated the ability to induce populations of cells with polyfunctional cytokine activity. The BCG/modified Vaccinia Ankara-Ag85A vaccine regime, for example, can generate CD4^+ ^T-cells that secrete up to 4 cytokines including IFNγ, TNFα, IL-2 and MIP-1β [19].

In the present study, we have used multiplex bead array to determine the concentration of 20 biomarkers (including cytokines and chemokines which together represent a comprehensive coverage of possible immune phenotypes) in the supernatants of *Mtb *PPD-stimulated, diluted whole blood assays on samples from UK adolescents taken prior to, and after BCG vaccination. A smaller panel of biomarkers were further investigated by flow cytometry. Cells from blood samples taken 12 months after BCG vaccination were stimulated and stained with antibodies to these biomarkers and other markers of T-cell phenotype.

## Results

### Biomarkers detected by diluted whole blood assay and multiplex bead array analysis

We set out to determine the characteristics of the profile of biomarkers present in peripheral blood from recently BCG vaccinated individuals (n = 12). Blood samples taken prior to and 1 month following BCG vaccination were diluted with RPMI 1640 and cultured for 6 days in the presence or absence of *Mtb *PPD. Assay supernatants were collected and frozen, then later analysed for biomarker content by multiplex bead array. The average sample storage time at ambient temperature between the collection of blood samples at schools and laboratory processing was 2.6 hours for both pre and 1 month post-vaccination samples.

**Figure 1:**
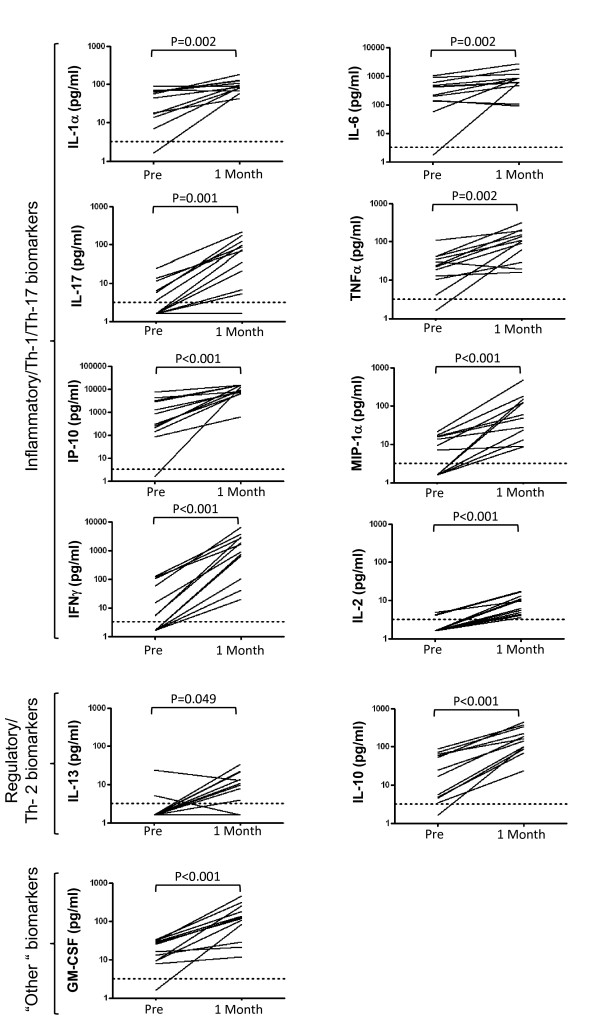
***Mtb *PPD-specific cytokine/chemokine biomarker responses 1 month post BCG vaccination of UK adolescents**. Plots representing concentrations of indicated biomarkers (see y-axis legends) are shown as measured by multiplex bead array assay on supernatants from 6 day diluted whole blood assays. Blood samples were donated from individuals (n = 12) prior to or 1 month following BCG vaccination. The dotted line indicates the lower limit of detection of the assay (3.2 pg/ml) and each data point represents the concentration measured in PPD-stimulated sample minus that measured in unstimulated sample. The Wilcoxon Signed Rank test was used to calculate p-values between pre and post-vaccination sample groups.

Of the 20 biomarkers that were measured, 10 were significantly raised (p < 0.0025) in assays carried out on samples taken 1 month after BCG vaccination (Figure 1 and Table 1). The majority of these were cytokines or chemokines associated with an inflammatory response (e.g. TNFα, IL-17, IL-1α, MIP1α, IL-6, IP-10) or the Th-1-type immune response (IFNγ, IL-2). Also increased were GM-CSF, and the anti-inflammatory cytokine IL-10. There was some evidence of an increase in IL-13 (p = 0.049) although increased stringency in testing due to multiple comparisons meant that this was not significant. There was no significant increase at 1 month post-vaccination in concentrations of IL-4 (p = 0.28), IL-8 (p = 0.09) or G-CSF (p = 0.18). Analytes that were undetectable in both pre and one month post vaccination samples were IL-12p70, IL-7, IL-15, IL-5, IL-1β and eotaxin (Table 1).

**Table 1:** Concentration (pg/ml) of 20 biomarkers measured prior to and 1 month after BCG vaccination of UK adolescents (values given are group median and interquartile range calculated after individual measurements were background corrected (PPD-stimulated - unstimulated values); n = 12)

Biomarker	Pre-vaccination (PPD-specific)	Pre-vaccination (background)	1 month post vaccination (PPD-specific) (*p < 0.0025)	1 month post-vaccination (background)
**IL-1α**	50.6 (15.2-64.6)	8.0	86.9 (73.4-116.6)*	11.5
**IL-1β**	1.6 (1.6-1.6)	1.6	1.6 (1.6-1.6)	1.6
**IL-2**	1.6 (1.6-2.8)	1.6	8.0 (4.6-12.1)*	1.6
**IL-4**	1.6 (1.6-1.8)	1.6	6.8 (1.6-40.1)	1.6
**IL-5**	1.6 (1.6-1.6)	1.6	1.6 (1.6-3.9)	1.6
**IL-6**	310.0 (140.5-537.7)	1.6	751.6 (521.8-1018.0)*	3.8
**IL-7**	1.6 (1.6-1.6)	1.6	1.6 (1.6-1.6)	1.6
**IL-8**	728.1 (656.4-5678.0)	58.5	7534.0 (4082.0-23400.0)	66.2
**IL-10**	20.8 (4.7-61.4)	1.6	153.8 (93.8-279.3)*	1.6
**IL-12p70**	1.6 (1.6-1.6)	1.6	1.6 (1.6-1.6)	1.6
**IL-13**	1.6 (1.6-1.6)	2.8	10.3 (2.8-17.6)	2.4
**IL-15**	1.6 (1.6-1.6)	1.6	1.6 (1.6-1.6)	1.6
**IL-17**	2.6 (1.6-9.0)	1.6	72.3 (14.1-111.7)*	1.6
**IFNγ**	5.3 (1.6-79.6)	1.6	1273.0 (373.8-2815.0)*	1.6
**TNF-α**	23.0 (11.7-38.3)	2.6	105.2 (45.8-176.3)*	1.6
**GM-CSF**	21.0 (9.4-29.5)	4.3	125.7 (56.8-216.6)*	5.9
**G-CSF**	1.6 (1.6-2.7)	1.6	3.6 (1.6-19.6)	1.6
**MIP-1α**	8.3 (1.6-16.0)	1.6	54.6 (18.5-138.8)*	1.6
**IP-10**	548.6 (169.5-2881.0)	33.1	8866.0 (6901.0-15000.0)*	52.4
**EOTAXIN**	1.6 (1.6-6.5)	34.1	1.6 (1.6-4.1)	8.8

Statistical comparisons were made using the Wilcoxon Signed Rank test. Median background concentration of each biomarker measured in unstimulated wells is also shown.

IFNγ is considered an essential component of the cytokine immune response to TB although not in itself sufficient to represent a correlate of protection. We were therefore interested in which biomarkers were associated with IFNγ responses in these assays (Table 2). Comparisons between the magnitude of cytokine responses as measured by concentration in assay supernatants revealed that there was a correlation between the magnitude of IFNγ responses and those of other Th-1 or pro-inflammatory biomarkers; TNFα (r = 0.91), MIP-1α (r = 0.87), IL-2 (r = 0.78), IL-17 (r = 0.77). There was also a strong correlation between IFNγ and the growth factor GM-CSF (r = 0.87) as well as with the anti-inflammatory cytokine IL-10 (r = 0.86). The weakest association with IFNγ responses was shown by IP-10 (r = 0.19).

**Table 2:** Correlation coefficients between the concentrations of IFNγ and other cytokines measured in whole blood assay supernatants one month after BCG vaccination

Biomarker	Spearman correlation with IFNγ responses (r)
TNFα	0.91
GM-CSF	0.87
MIP-1α	0.87
IL-10	0.86
IL-2	0.78
IL-17	0.77
IL-6	0.69
IL-1α	0.56
IL-13	0.55
IP-10	0.19

We next investigated whether biomarkers of interest detected by multiplex early after BCG vaccination were produced in response to stimulation at 12 months post-vaccination. Due to loss to follow up at 12 months of some individuals included in the pre-vaccination and 1 month analysis, the 12 samples analysed by multiplex at 12 months only partly overlap with the group analysed at the earlier time point. They did however match the 12 month PBMC samples that were available for flow cytometric analysis (see below). Using diluted, 6 day whole blood assays with *Mtb *PPD stimulation and multiplex bead array analysis a broad array of biomarkers remained detectable with median responses above the assay's lower limit of detection of 3.2 pg/ml (Table 3). Eight of the ten biomarkers that were significantly raised in 1 month post-BCG vaccination samples remained raised in 12 month samples. IL-6 and IL-10 returned to levels that were comparable to those measured in pre-vaccination samples. Unfortunately, following a company takeover and the subsequent discontinuation of the original Lincoplex kit, it was necessary to use Millipore's new Milliplex version of the multiplex assay for 12 month sample analysis. Due to some evidence of an increase in sensitivity for some analytes and a decreased sensitivity for others, measured using the Milliplex kit compared to the Lincoplex kit (data not shown), we did not consider it valid to draw statistical comparisons between data collected using the two kits. The 12 month data demonstrate however that, within the parameters of the kit used, a broad array of biomarkers remain detectable.

**Table 3:** Concentration (pg/ml) of 20 biomarkers measured 12 months after BCG vaccination of UK adolescents (values given are group median and interquartile range calculated after individual measurements were background corrected (PPD-stimulated - unstimulated values); n = 12)

Biomarker	12 months post vaccination
**IL-1α**	1710.0 (949.2-2008.0)
**IL-1β**	4.5 (1.6-10.1)
**IL-2**	8.2 (5.3-14.8)
**IL-4**	1.6 (1.6-1.8)
**IL-5**	1.6 (1.6-4.7)
**IL-6**	383.0 (198.7-1653.0)
**IL-7**	48.4 (36.2-95.6)
**IL-8**	6030.0 (521.8-11935.0)
**IL-10**	12.7 (10.2-22.2)
**IL-12p70**	1.9 (1.6-5.9)
**IL-13**	1.6 (1.6-6.9)
**IL-15**	1.6 (1.6-1.6)
**IL-17**	22.0 (3.0-37.8)
**IFNγ**	79.0 (37.9-239.1)
**TNF-α**	38.9 (26.9-140.0)
**GM-CSF**	147.7 (88.7-289.0)
**G-CSF**	3.8 (1.6-10.0)
**MIP-1α**	69.9 (27.0-279.3)
**IP-10**	4240.0 (2645.0-7670.0)
**EOTAXIN**	29.9 (23.7-37.1)

We conclude therefore, that BCG vaccination results in a broad biomarker response measurable at one month in peripheral blood that is detectable following *Mtb *PPD re-stimulation. Furthermore, much of this broad response is maintained until at least 12 months following vaccination.

### Flow cytometric analysis of cellular cytokine production

At this stage, we utilised additional material available from blood samples taken at the 12 month post-BCG time point when PBMC were isolated and cryopreserved (n = 12). Recovered cells were stimulated for 24 hours in the presence or absence of *Mtb *PPD (plus Brefeldin A for the final 6 hours) and stained with a panel of antibodies recognising phenotypic lymphocyte markers and the cytokines IFNγ, TNFα, IL-2, IL-17 and IL-10 before acquisition.

**Figure 2:**
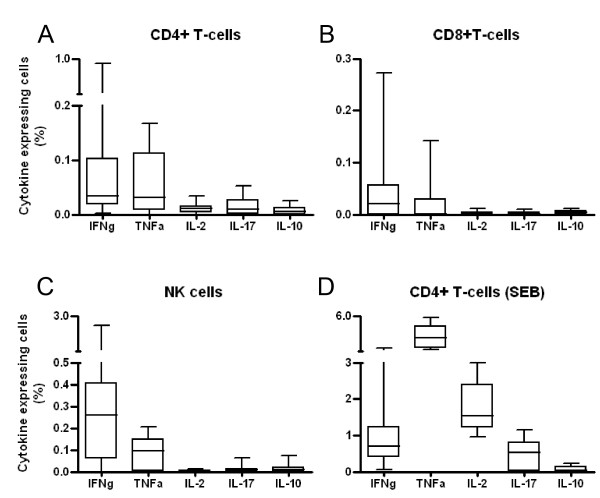
***Mtb *PPD-specific cytokine expression in CD4 T-cells, CD8 T-cells and NK cells 12 months post BCG vaccination of UK adolescents**. Box plots representing the proportion of A) CD4 T-cells, B) CD8 T-cells and C) NK cells that express the indicated cytokine (see x-axis labels) are shown as determined by intracellular cytokine staining of *Mtb *PPD-stimulated PBMC. D) CD4 T-cell cytokine expression in response to the positive control stimulant SEB. Cells were isolated from blood samples from individuals (n = 12) 12 months following BCG vaccination. The horizontal line represents the median response, the box represents the interquartile range and the whiskers represent the overall range.

Following gating on singlet and live cells and the exclusion of CD14 or CD19-expressing cells, single cytokine expression was examined in CD4 T-cells (CD3^+^CD4^+ ^subset), CD8 T-cells (CD3^+^CD8^+ ^subset) and natural killer (NK) cells (CD3^-^CD56^+ ^subset) (Figure 2A-C). IFNγ expression was detected in all three subsets examined, the median IFNγ-expressing proportion of each subset being 0.03% (CD4 T-cells), 0.02% (CD8 T-cells) and 0.26% (NK cells). TNFα expression was detected in CD4 T-cells (0.03%) and NK cells (0.1%) however the median proportion of CD8 T-cells expressing TNFα was very low (<0.01%). Similarly, median expression levels of IL-2, IL-17 and IL-10 after *Mtb *PPD stimulation were all less than 0.01%. Stimulation of cells with the positive control Staphylococcus enterotoxin B (SEB) produced detectable expression of IFNγ, TNFα, IL-2 and IL-17 but not IL-10 (Figure 2D).

**Figure 3:**
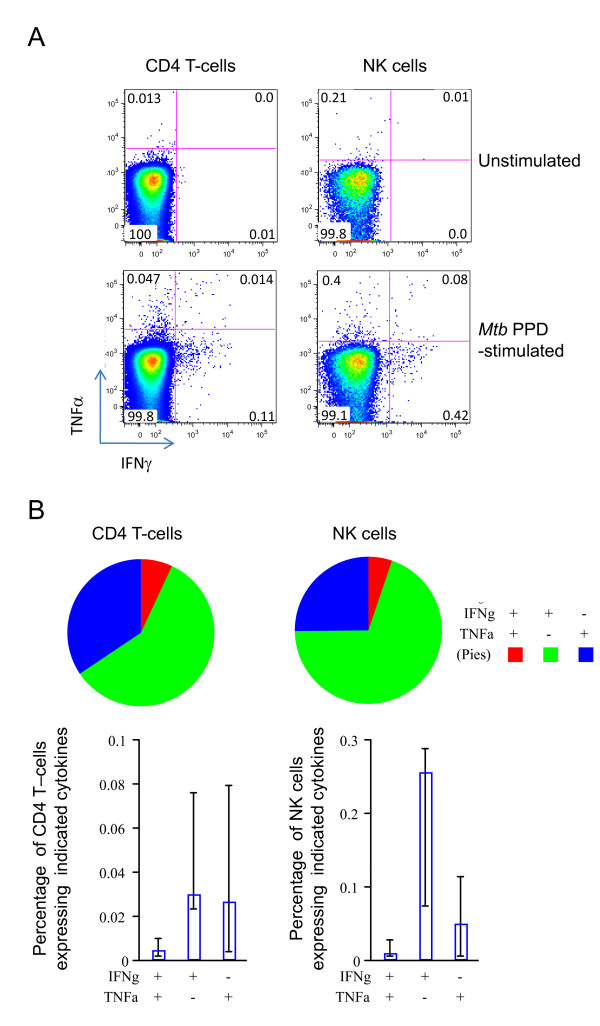
**Polyfunctional analysis of *Mtb *PPD-specific cytokine expression in CD4 T-cells and NK cells 12 months post BCG vaccination of UK adolescents**. A) Dot plots from one individual donor are shown comparing simultaneous expression of IFNγ (x-axis) and TNFα (y-axis). Previous gating was used to isolate singlet, live cells that were either CD3^+^CD4^+ ^(CD4 T-cells) or CD3^-^CD56^+ ^(NK cells) as indicated for each plot. Responses in unstimulated and *Mtb *PPD-stimulated samples are shown. Values indicate the proportion (as a percentage) of the indicated subset present in that quadrant. B) Boolean gating and Spice analysis was used to determine the extent of polyfunctionality within the 12 month post-BCG vaccination group (n = 12). Bar charts represent, for either CD4 T-cells or NK cells, the median percentage (and interquartile range) of each subset expressing the indicated cytokine combination (x-axis labels). Pie segments represent the average proportion, across the group, of cells expressing the indicated cytokine combination (pie chart legend) within the total population of cells expressing either IFNγ or TNFα.

Having detected both IFNγ and TNFα expression separately in the CD4 T-cell and NK cell subsets we next assessed to what extent expression of these two biomarkers was to be found in single, polyfunctional responders (Figure 3). Although dot plots revealed that a small number of IFNγ and TNFα double positive CD4 T-cells and NK cells were present in some individuals (Figure 3A), the proportions of all CD4 T-cells or NK cells expressing either cytokine that were in fact double positive in the whole group was small (Figure 3B). The majority of cytokine producing cells were only producing either IFNγ or TNFα. For CD4 T-cells and to a greater extent NK cells, IFNγ single positive cells comprised the largest population.

Taken together, these data demonstrate that in this sample set, multiple cytokine responses are less readily detectable by flow cytometry compared to the multiplex bead array analysis involving a six day culture. Where both *Mtb *PPD-specific IFNγ and TNFα expression was detected in CD4 T-cells and NK cells it was mostly in single cytokine producers rather than in cells of dual function.

## Discussion

The cytokine response measured following BCG vaccination of adolescents in this study was broad. Such a varied response was also observed by us and others in infant studies of BCG vaccination [17,18]. Of 20 analytes measured, 10 were significantly raised in *Mtb *PPD stimulated, diluted whole blood samples taken 1 month after BCG vaccination compared to pre-vaccination samples. IFNγ was the most markedly increased analyte (fold increase of 240 in post-vaccination samples), which bears out this cytokine's long-standing association with mycobacterial immunity [2,20]. TNFα and IL-2 have also both been described by others as cytokines that are central to effective immunity to TB [5,16] and indeed we found them to be raised following BCG (increased by a factor of 4.6 and 5.0 respectively). The protection that has been afforded against TB by BCG vaccination in the UK is likely to rely on the actions of these cytokines. IFNγ and TNFα can activate macrophages to better destroy phagocytosed *Mtb *[21] and TNFα helps induce maturation of dendritic cells for more effective activation of mycobacteria-specific T-cells [22]. IL-2 is also responsible for the promotion of T-cell responses and memory [23]. Such immune functions rely on the targeted release of these cytokines by antigen experienced effector cells. That BCG vaccination is priming such cells is indicated by our observation that these biomarkers are detected at increased concentrations in post-vaccination samples after stimulation with *Mtb *PPD. The many other pro-inflammatory biomarkers detected in post-vaccination assay supernatants may represent a combined response originating from Th-1 cells, blood monocytes or neutrophils that are responding to IFNγ and/or TNFα. It should be noted that the inflammatory biomarkers IL-6, IL-8 and IP-10, although increased in post-vaccination samples were also present in relatively large amounts in pre-vaccination samples following *Mtb *PPD stimulation. This suggests an antigen-non-specific response of the innate immune system, possibly as a result of stimulation of monocytes or neutrophils via toll-like receptors by an undefined component of the *Mtb *PPD protein cocktail. This hypothesis requires further investigation. In addition, IL-8 and IP-10 were present in unstimulated wells to a greater extent than other biomarkers suggesting that these are released, to some degree in these conditions, spontaneously.

IL-17 is a pro-inflammatory cytokine that has been linked to a number of autoimmune and chronic inflammatory conditions such as rheumatoid arthritis [24] and asthma [25]. In mice, γδ T-cell-derived IL-17 has been demonstrated during *Mtb *and BCG infection and in the latter, shown to contribute to effective Th-1 responses and granuloma formation [26,27]. In our current study, IL-17 was strongly increased in post-BCG whole blood assay supernatants (28.3 fold increase) and was also significantly associated with concurrent increases in IFNγ (r = 0.769). As *Mtb *PPD comprises a number of undefined components these results do not determine the precise nature of the IL-17 response detected. Possibilities include a CD4^+ ^Th-17 response to protein antigen(s) in *Mtb *PPD that are processed and presented by MHC class II molecules. Alternatively, as there is evidence from other studies that γδ T-cells may produce IL-17, it may be that in our assay such cells are recognising an unknown component of *Mtb *PPD.

If BCG vaccination of UK adolescents is taken as a setting where there has been a degree of success in promoting immunity to TB, it is surprising to see the anti-inflammatory cytokine IL-10 also raised in *Mtb *PPD-stimulated, post-BCG assay supernatants. It is possible that the 6 day duration of the whole blood assay may allow time for a complex network of physiological homeostatic interactions between the cells present and the cytokines released on stimulation with *Mtb *PPD. One outcome of this may be the initiation of regulatory negative feedback loops that are characterised by the production of IL-10.

Any biomarkers released upon an encounter with *Mtb *antigen may originate from specific lymphocyte populations activated by vaccination. Although multiplex bead array assays are a powerful tool, they cannot identify the cellular source of any biomarkers measured. For this reason we selected a smaller panel of biomarkers (IFNγ, TNFα, IL-2, IL-17, IL-10) that could be assessed by flow cytometry using specific monoclonal antibodies. When combined with antibodies to phenotypic markers it was possible to identify the cells that produced certain cytokines. Due to availability, only PBMC from 12 month post-BCG samples were available for flow cytometric analysis. However, we used the multiplex assay to demonstrate that many of the biomarker responses detectable at one month remained detectable at 12 months.

In this study of *Mtb*-specific memory lymphocytes induced by BCG, we detected predominantly single cytokine producing cells when PBMC were stimulated for 24 hours with *Mtb *PPD. The largest responder phenotype in each PBMC sub-population (CD4 T-cells, CD8 T-cells and NK cells) was IFNγ single positive cells. NK cells and CD4 T-cells also contained populations of TNFα single positive responding cells, but double positive cells were less common. For the CD8 T-cell population, single positive IFNγ cytokine secreting cells represented the only notable responding cells. In a study of infant responses to BCG vaccination from a TB-endemic country, analysis of live BCG-stimulated whole blood samples showed that, as in our study, within the CD8^+ ^T-cell population, the dominant population expressed IFNγ only [17]. However, three other secretion patterns were also detected within the CD8^+ ^T-cells in that study; IL-2 only, IFNγ/TNFα/IL-2 triple positive and IFNγ/IL-2 double positive cells although these latter three patterns were at levels only slightly above background. Such responses may be indicative of the superior ability of live BCG over *Mtb *PPD to stimulate CD8 T-cells as previously reported [28]. Also, in contrast to our study, Soares *et al *detected 7 distinct patterns of cytokine secretion within the CD4^+ ^T-cell population including triple, double and single positive cytokine producers whereas we only observed 2 (IFNγ and TNFα single positives). Using a PBMC stimulation method similar to that described here, polyfunctional T-cells (secreting up to four cytokines including IFNγ, TNFα and IL-2) have been demonstrated following Modified Vaccinia Ankara-85A administration after previous BCG vaccination of a UK cohort [19]. The lack of multiple cytokine secreting cells in our study may be a result of the late timing of these samples (12 months post-vaccination). Any polyfunctional, memory lymphocytes present may exist only at very low frequencies that would require the acquisition of many more events than were possible in this study due to the amount of sample available. Another possibility is that the exposure of responding lymphocytes to antigen is minimal or absent 12 months after BCG vaccination. Any multifunctional T-cells may, as a result, convert to a resting state out of which the short incubation time we used in flow cytometry assays is insufficient to stimulate them. It should also be considered however, that the failure of BCG to deliver long-lasting (i.e. lifetime) immunity against pulmonary TB in many settings may be predicted by the lack of induction of long term polyfunctional T-cell responses as shown here. Unfortunately, the lack of pre-vaccination or 1 month post-vaccination comparator samples for the flow cytometry aspect of this study is a significant limitation and renders us unable to comment on the absolute effect of BCG vaccination on intracellular cytokine profiles. We were unable to detect any expression of IL-2, IL-17 or IL-10 by flow cytometry after *Mtb *PPD stimulation. Again, this may be explained by the timing of the samples and the need to acquire a large number of events to detect very rare, cytokine producing cells. Soares *et al. *also reported low percentages of IL-10 expressing T-cells that were, in most cases, below their cutoff for a positive response [17]. The stimulation period used in that study (12 hours) and in ours (24 hours) may be sub-optimal for the detection of IL-10. Although we did not detect IL-17 after *Mtb *PPD stimulation, others have observed CD3^+^CD4^+^IL-17^+ ^cells following BCG and PPD stimulation of whole blood from mycobacteria-exposed individuals [29]. IL-17 induced by *Mtb *PPD was reported to be low by Scriba *et al *and our use of purified PBMC as opposed to their use of whole blood and the inherent antigen processing differences involved may explain the absence of IL-17^+ ^cells in the current study. Others have also found that purified T-cells with specificity for *Mtb *PPD produced IFNγ but failed to produce IL-17 in response to stimulation [30]. It should be noted that at 72 pg/ml, the median concentration of IL-17 measured by multiplex bead array is relatively low in 1 month post-BCG whole blood assay supernatants compared to IFNγ (1273 pg/ml) and the increase over the median concentration in pre-vaccination assays is only detectable due to the sensitivity of the multiplex assay. Such marginal secretion kinetics may be less easy to observe using a 24 hour flow cytometry assay at 12 months post BCG.

## Conclusions

In summary, we have shown that the *Mtb *PPD-specific response 1 and 12 months after BCG vaccination as measured in a 6 day, diluted whole blood assay was broad in terms of cytokines released and encompassed Th-1 and inflammatory mediators as well as immunoregulatory elements. Such a multi-faceted response indicates the complex nature of the immune response to BCG as well as the ability to detect such responses in longer duration assays. When *Mtb *PPD-stimulated PBMC were analysed by flow cytometry, polyfunctional CD8 or CD4 T-cells were not detected. The majority of responding cells in these populations and especially in the NK cell population were single cytokine (either IFNγ or TNFα) producers. Although these findings may relate to sample sizes, timings and assay kinetics they may also represent an inability of BCG to induce the polyfunctional T-cells that are thought to be important mediators of immunity against infection [31]. Whether such cells are detectable earlier following vaccination and are lost at later time points remains to be seen.

## Methods

### Subjects

Informed, written consent was obtained from the parents of healthy UK adolescents due to receive tuberculin (Heaf) skin testing and BCG vaccination as part of the UK schools BCG programme. Verbal consent was given by volunteers and ethical approval was obtained from the Redbridge and Waltham Forest National Health Service Local Research Ethics Committee and from the London School of Hygiene & Tropical Medicine internal Ethics Committee. Year 8 adolescents (12-13 years old) were invited to take part and exclusion was based on evidence previous of BCG vaccination (BCG scar or subsequent positive tuberculin skin test).

### Blood samples

Ten mL venous blood samples were taken from adolescent study participants (subject to availability for venepuncture) prior to tuberculin skin testing and again at 1 month (the peak of the PPD-specific response following BCG vaccination [32]) and 12 months after vaccination with BCG-Danish (Statens Serum Institute, Copenhagen). Blood samples were transferred to 15 mL centrifuge tubes (Greiner, Germany) containing 100 units of preservative free heparin sodium (Monoparin, CP Pharmaceuticals, UK). These were stored at room temperature for transportation to the laboratory.

### Diluted whole blood assay

Venous blood was diluted 1 in 10 in RPMI 1640 medium (Invitrogen, UK) containing 2 mM L-glutamine (Invitrogen, UK) and cultured in 96-well U-bottomed tissue culture plates (Corning, USA) in a final volume of 200 μL with or without stimulation. Antigen *Mtb *PPD (Statens Serum Institute, Denmark)) was used at a concentration of 5 μg/mL. Cultures were incubated for 6 days in humidified incubators at 37°C, 5% CO_2 _after which culture supernatants were collected and stored at -80°C.

### Multiplex bead array

Twenty-one cytokines and chemokines were measured simultaneously in culture supernatant from assays carried out on samples obtained prior to and 1 month after BCG vaccination using a human cytokine Lincoplex premixed kit according to the manufacturer's instructions (cat #HCYTO-60K-PMX, Linco Research Inc, St Charles Missouri, USA): IL-1β, IL-2, IL-4, IL-5, IL-6, IL-7, IL-8, IL-10, IL-12p70, IL-13, IL-15, IL-17, IL-1α, IFNγ, G-CSF, GM-CSF, TNFα, Eotaxin, MCP-1, MIP1α and IFNγ inducible protein (IP)-10. Unstimulated and *Mtb *PPD stimulated samples were read on the Biorad Luminex reader using Bioplex manager 4.1 software. For each cytokine the standard curve ran from 3.2 to 10,000 pg/ml. Supernatants collected from assays carried out on samples obtained 12 months after BCG vaccination were tested on a separate occasion using a human cytokine/chemokine Milliplex™ MAP premixed kit according to the manufacturer's instructions (cat #MPXHCYTO-60K-PMX-42, Millipore Corp, St Charles Missouri, USA).

### PBMC preparation and cryopreservation

PBMC were isolated from venous blood samples obtained 12 months post-BCG vaccination. Blood was added to 50 mL Leucosep tubes (Greiner, Germany) containing 15 mL Histopaque 1077 (Sigma, UK). Tubes were centrifuged for 10 minutes at 1000 *g*. The PBMC layer was transferred into fresh 15 mL centrifuge tubes, washed 3 times in HBSS (Invitrogen, UK) and cells counted. PBMC were frozen at 5 × 10^6 ^cells per ml of cryopreservation medium (45% RPMI 1640, 45% FBS, 10% DMSO) using a Mr Frosty™ container (Nalgene) overnight at -80°C before transfer to liquid nitrogen.

### Intracellular cytokine staining

PBMC were thawed and incubated for 10 minutes at 37°C in AIM-V medium (Invitrogen, UK) containing 10 units/ml DNAse (Sigma), then washed and resuspended at 10^6 ^cells per ml in AIM-V. Depending on availability, between 5 × 10^5 ^and 10^6 ^cells were incubated for 24 hours in 15 ml polypropylene centrifuge tubes at 37°C with either medium alone, *Mtb *PPD at 10 μg/ml or positive control Staphylococcus enterotoxin B (SEB; Sigma) at 1 μg/ml. Brefeldin A was added to a concentration of 10 μg/ml for the final 6 hours of incubation. The contents of each centrifuge tube were transferred to 2.5 ml polystyrene flow cytometer tubes (Falcon), washed with PBS containing 1% BSA and 0.1% sodium azide and stained with the VIVID live/dead reagent (Molecular Probes) and conjugated monoclonal antibodies: anti-CD3-AmCyan; anti-CD56-PE-Cy5 (BD Biosciences); anti-CD4-Qdot^®^605 (Invitrogen); anti-CD8-APC-Alexa Fluor^®^750; anti-CD14-PacificBlue (Caltag Laboratories) and anti-CD19-PacificBlue (eBiosciences). After further washing and permeabilisation with Cytofix/cytoperm reagent (BD Biosciences), cells were further stained with conjugated monoclonal antibodies: anti-IFNγ-Alexa Fluor^®^700 (BD Biosciences); anti-TNFα, PE-Cy7; anti-IL-2-APC; anti-IL-17-PE (eBiosciences) and anti-IL-10-FITC (Caltag Laboratories). Cells were finally washed and fixed with 1% paraformaldehyde before acquisition using an LSRII flow cytometer (Becton Dickinson) and FACSdiva software. Compensation was achieved using BD™ CompBeads (BD Biosciences). Between 2 × 10^5 ^and 6 × 10^5 ^total events (mean 3.5 × 10^5^) were acquired per sample (CD3^+^CD4^+ ^mean acquired events - 9 × 10^4^; CD3^+^CD8^+ ^mean acquired events - 4 × 10^4^; CD3^-^CD56^+ ^mean acquired events - 3 × 10^4^). Collected data was analysed using FlowJo software (Version 8.8.1; Tree Star Inc.) and the data analysis programs Pestle (version 1.6.1) and Simplified Presentation of Incredibly Complex Evaluations (SPICE, version 4.3) kindly provided by Mario Roederer, Vaccine Research Center, NIAID, NIH.

### Data analysis

For multiplex bead array assays, for each donor, biomarker concentrations measured in unstimulated assays were subtracted from those measured in *Mtb *PPD stimulated assays. One in ten dilutions of supernatants were also tested in some cases and these corrected values were used when neat samples were beyond the assay's upper limit of detection. Corrected values that were less than the assay's lower limit of detection (3.2 pg/ml) were allocated a value half this amount of 1.6 pg/ml representing an undetectable response as previously described [18,33,34]. For samples where the concentration of a particular biomarker was above the limit of detection, values were assigned based on the highest detectable concentration for that biomarker in other samples (30,000 pg/ml for IL-8 and 15,000 pg/ml for IP-10). Due to a large number of samples with unreadable MCP-1 concentrations, this biomarker was omitted from further analyses.

For flow cytometry, CD19^+ ^(B-cells) and CD14^+ ^(monocytes) events appeared in the same channel as VIVID live/dead positive events (dead cells) and were together negatively gated out of the analysis. Remaining CD3^+^CD4^+ ^(CD4 T-cells), CD3^+^CD8^+ ^(CD8 T-cells) and CD3^-^CD56^+ ^(NK cells) events were analysed for cytokine expression. Expression levels in unstimulated samples were subtracted from those measured in *Mtb *PPD-stimulated samples. The Boolean gate function of FlowJo was used to create outputs for all possible cytokine expression combinations using individual cytokine analysis gates described above.

### Statistical analysis

The Wilcoxon Signed Rank test was used to compare cytokine/chemokine responses in paired samples from pre-vaccination and 1 month post vaccination time points. The test for significance was set at p < 0.0025 to allow for multiple comparisons. Spearman's rank correlation coefficient was calculated to compare the association between IFNγ and other biomarkers measured 1 month after vaccination.

## Abbreviations

TB: tuberculosis; *Mtb *PPD: *Mycobacterium tuberculosis *purified protein derivative; IFNγ inducible protein-10 (IP-10)

## Authors' contributions

SGS co-ordinated and participated in the study design, performed the experiments and drafted the manuscript. ML, PGS and RB participated in sample collection and processing and/or immunoassays. NERB, AW and HM participated in the study design regarding flow cytometry. HMD conceived of the study, and participated in its design and coordination. All authors read and approved the final manuscript.
